# Knowledge, attitude, and practice of cancer patients regarding radiotherapy and radiation protection

**DOI:** 10.3389/fonc.2025.1432187

**Published:** 2025-07-30

**Authors:** Di Cui, Peng Wang, Lei Kong, Jidong Wang, Chuanhao Tang, Jun Liang

**Affiliations:** ^1^ Department of Radiation Oncology, Peking University International Hospital, Beijing, China; ^2^ Department of Oncology, Peking University International Hospital, Beijing, China

**Keywords:** knowledge, attitude, practice, cancer patient, radiotherapy, radiation protection, cross-sectional study

## Abstract

**Introduction:**

This study aimed to investigate the knowledge, attitude, and practice (KAP) of cancer patients regarding radiotherapy and radiation protection.

**Methods:**

A cross-sectional study was conducted between December 2023 and January 2024, at Peking University International Hospital, and included cancer patients through convenience sampling. Demographic characteristics and KAP scores were collected through distributed questionnaires.

**Results:**

A total of 497 valid questionnaires were collected, with 252 (50.70%) completed by females. Of the respondents, 463 (93.16%) demonstrated awareness of the irradiation site for their treatment. Mean knowledge, attitude, and practice scores were 14.84 ± 4.99 (possible range: 0 - 28), 26.94 ± 3.63 (possible range: 8 - 40), and 25.24 ± 4.26 (possible range: 6 - 30), respectively. Correlation analyses revealed significant positive correlations between knowledge and attitude (r = 0.141, P = 0.002), as well as practice (r = 0.300, P < 0.001). Similarly, there was a correlation between attitude and practice (r = 0.279, P < 0.001). The path analysis revealed that knowledge was significantly associated with attitude (β = 0.142, P < 0.001) and practice (β = 0.202, P < 0.001). Similarly, attitude was associated with practice (β = 0.310, P < 0.001).

**Conclusion:**

Cancer patients exhibited inadequate knowledge, moderate attitudes and proactive practices towards radiotherapy and radiation protection. The positive correlations between knowledge and various factors, such as radiotherapy awareness, ionizing radiation understanding, and patient comprehensive scores, underscore the integral role of patient education in enhancing their approach to radiotherapy and radiation protection.

## Introduction

Cancer, a multifaceted disease marked by the uncontrolled proliferation of abnormal cells, presents a substantial global health challenge (1). Among the array of treatment modalities, including immunotherapy, chemotherapy, surgery, and radiotherapy, the latter plays a pivotal role. Its significance is underscored by its applicability to over 50% of all cancer patients, emphasizing its profound impact on cancer management (2). Radiotherapy, as a treatment modality, employs high doses of radiation to precisely target and eliminate cancer cells while minimizing damage to adjacent healthy tissues (3). Crucially, it can be administered either independently or in conjunction with other interventions such as surgery and chemotherapy (4). Despite its critical role in cancer management, radiotherapy carries inherent risks of radiation-induced damage to normal tissues, giving rise to potential short-term and long-term complications (5). One noteworthy example of such complications is radiation-induced lung injury, particularly prevalent in patients undergoing radiotherapy for chest tumors (6). Consequently, the medical application of ionizing radiation necessitates continuous research and advancements in radiation protection (7).

The Knowledge Attitude Practice (KAP) theory plays a pivotal role in shaping human health behaviors and holds particular significance in the realm of health literacy (8). This model operates on the fundamental principle that knowledge positively influences attitudes, which in turn shape practices (9). Within the healthcare domain, the KAP questionnaire serves as a vital tool for comprehensive assessment, delving into the knowledge, attitudes, and practices of a specific population. It allows for the evaluation of their understanding, perceptions, and behaviors, while also gauging the demand for and acceptance level of relevant health-related information (10). This approach gains particular relevance in the context of cancer treatment, where the KAP survey method provides invaluable insights into the educational needs and intervention strategies for cancer patients. Despite the widespread use of radiotherapy in cancer care, patients often exhibit notable knowledge gaps, especially concerning radiation-related aspects. This deficiency in understanding can significantly impact their treatment experiences and outcomes. Inadequate knowledge about the nature and safety of radiotherapy may elevate patient anxieties, foster misconceptions, and potentially lead to diminished adherence to treatment protocols (11). Therefore, a comprehensive understanding of KAP specific to radiotherapy and radiation protection becomes essential in bridging these gaps. However, a considerable void currently exists in KAP research specifically targeting this area, emphasizing the urgent need for more focused studies in this field.

Therefore, this study aims to investigate the cancer patients’ KAP towards radiotherapy and radiation protection.

## Methods

### Study design and participants

This cross-sectional study was conducted at Peking University International Hospital between December 2023 and January 2024, and included cancer patients by convenience sampling. The inclusion criteria were: 1) aged over 18 years old; 2) were undergoing or had undergone radiotherapy; 3) A Performance Status (PS) score of 0-1. The exclusion criteria were: 1) Refusal to participate; 2) impaired cognitive function due to brain metastasis or other conditions. This study was ethically approved by the Ethics Committee of Peking University International Hospital, and informed consent was obtained from all participants.

The questionnaires for this study were disseminated using *“Wenjuanxing (* *http://www.wjx.cn* *)”*, a widely utilized online survey platform in China. Participants accessed the questionnaire and scales by scanning QR codes linked to Wenjuanxing, facilitating an efficient and user-friendly means of data collection.

### Questionnaire

The design of the questionnaire was grounded in prior studies (12–14), and it was refined based on feedback from 15 experts, including 3 radiologists, 4 oncologists, 6 medical professionals, and 2 public health experts. Subsequently, a pilot test with 30 participants was carried out, yielding a Cronbach’s α = 0.899, indicating a good internal consistency.

The final questionnaire, presented in Chinese, is structured into 6 sections: demographic characteristics, knowledge, attitude, practice, medical information acquisition, and health literacy. And a comprehensive patient score was assessed by healthcare professionals to evaluate the patients’ practice from the medical perspective. The knowledge section is further divided into “Radiotherapy and Radiation Protection” and “Ionizing Radiation.” The former comprises 7 questions, scored as 2 for “well-known,” 1 for “heard of,” and 0 for “unknown,” totaling a range of 0–14 points. The latter section assigns 2 points for correct answers and 0 points for incorrect or unclear responses, also totaling 0–14 points, with the overall knowledge section ranging from 0–28 points. The attitude section includes 9 questions on a five-point Likert scale, from very positive (5 points) to very negative (1 point), and the question 9 was not scored because it does not reflect positive or negative attitude, spanning a range of 8–40 points. The medical information acquisition and health literacy dimensions each consist of 3 questions, also on a five-point Likert scale, assessing positively oriented behavior from always (5 points) to never (1 point), with both sections having a range of 3–15 points (15). The practice dimension contains 6 questions, evaluated similarly on a five-point Likert scale, with a range of 6–30 points. The patient score, a comprehensive measure self-administered by individuals and evaluated by healthcare professionals, spans a range of 0–8 points. Participants scoring above 80% of the total in each section were categorized as having adequate knowledge, a positive attitude, and proactive practice. Those scoring between 60% and 80% were classified as having moderate knowledge, attitude, and practice. Scores below 60% indicated inadequate knowledge, a negative attitude, and inactive practice (16).

### Statistical analysis

SPSS 26.0 (IBM, Armonk, NY, USA) and AMOS (IBM, Armonk, NY, USA) were used for statistical analysis. The continuous data were presented as mean ± standard deviation (SD), and compared by the Mann-Whitney test and Kruskal-Wallis H test. The categorical data were described using n (%). The Spearman’s correlation analysis was utilized to assess the correlations among the dimensions. Path analysis was employed to explore the interactions among KAP. A two-sided P < 0.05 was considered as statistical significance.

## Results

Initially, a total of 500 individuals participated in the study, with 3 were excluded due to conflict response, resulting in 497 valid questionnaires, with a validity rate of 99.4%. Among them, 252 (50.70%) were female, 249 (50.10%) were not more than 52 years old, and 136 (27.36%) graduated from junior high school and below. In addition, 108 (21.73%) had a family number with medical education background, 448 (90.14%) lived with others, 463 (93.16%) were aware of the site of irradiation for this treatment, 350 (70.42%) were aware of the type of radiotherapy for this treatment, and 396 (79.68%) had a health literacy score of no more than 9 points (**Table 1**).

**Table 1:** Demographic characteristics, KAP scores, medical information acquisition, and patient comprehensive score.

Characteristics	N (%)	Knowledge		Attitude		Practice		Medical information acquisition		Patient comprehensive score	
		Score	P	Score	P	Score	P	Score	P	Score	P
Total	497 (100.00)	14.84 ± 4.99		26.94 ± 3.63		25.24 ± 4.26		10.51 ± 1.37		7.83 ± 0.46	
Gender			0.570		0.990		0.307		0.976		0.767
Male	245 (49.30)	15.09 ± 4.99		26.98 ± 3.71		25.51 ± 4.23		10.47 ± 1.30		7.85 ± 0.42	
Female	252 (50.70)	14.60 ± 4.99		26.90 ± 3.56		24.98 ± 4.28		10.54 ± 1.45		7.81 ± 0.49	
Age, years	50.89 ± 15.36		0.027		0.864		0.541		0.170		0.616
≤52	249 (50.10)	15.48 ± 5.27		26.97 ± 3.79		25.47 ± 4.13		10.63 ± 1.38		7.82 ± 0.46	
>52	248 (49.90)	14.20 ± 4.61		26.92 ± 3.48		25.00 ± 4.39		10.38 ± 1.36		7.85 ± 0.45	
Residence			0.561		0.052		<0.001		0.011		0.887
Rural	175 (35.21)	14.55 ± 5.16		26.65 ± 3.49		24.07 ± 4.66		10.25 ± 1.27		7.81 ± 0.51	
Urban	283 (56.94)	15.06 ± 4.90		27.26 ± 3.73		25.93 ± 3.83		10.68 ± 1.44		7.85 ± 0.43	
Suburban	39 (7.85)	14.56 ± 4.87		25.95 ± 3.38		25.44 ± 4.36		10.44 ± 1.19		7.85 ± 0.37	
Education			<0.001		0.006		0.002		0.011		0.565
Junior high school and below	136 (27.36)	14.11 ± 5.04		27.02 ± 3.65		24.50 ± 4.51		10.26 ± 1.37		7.831 ± 0.46	
High school and technical secondary school	124 (24.95)	13.96 ± 4.76		26.01 ± 2.88		24.65 ± 4.07		10.37 ± 1.21		7.855 ± 0.42	
College	95 (19.11)	15.04 ± 5.08		28.05 ± 3.81		25.58 ± 4.03		10.68 ± 1.65		7.768 ± 0.55	
Bachelor’s degree	109 (21.93)	15.72 ± 4.87		26.80 ± 3.71		26.17 ± 4.18		10.70 ± 1.27		7.84 ± 0.44	
Master’s degree and above	33 (6.64)	17.67 ± 4.40		27.39 ± 4.47		26.48 ± 4.12		10.88 ± 1.29		7.94 ± 0.24	
Employment			<0.001		0.532		<0.001		0.112		0.574
Full-time	139 (27.97)	16.55 ± 4.63		27.19 ± 3.72		26.32 ± 3.72		10.72 ± 1.37		7.84 ± 0.44	
Part-time/Self-employed/Freelancer	98 (19.72)	13.89 ± 5.02		27.12 ± 3.63		24.80 ± 4.10		10.49 ± 1.30		7.86 ± 0.41	
Unemployed/Laid off	55 (11.07)	13.38 ± 4.34		26.27 ± 3.33		23.16 ± 4.47		10.09 ± 1.48		7.84 ± 0.46	
Full-time housewife/husband	35 (7.04)	14.20 ± 5.34		26.71 ± 3.43		23.66 ± 4.81		10.20 ± 1.62		7.86 ± 0.43	
Retired	138 (27.77)	14.28 ± 4.83		26.99 ± 3.56		25.68 ± 4.04		10.53 ± 1.31		7.83 ± 0.50	
Student	32 (6.44)	15.97 ± 5.83		26.50 ± 4.37		25.31 ± 5.21		10.59 ± 1.27		7.72 ± 0.52	
Monthly Income, Yuan			0.006		0.537		0.002		0.063		0.272
<2000	84 (16.90)	13.63 ± 5.05		27.13 ± 3.68		24.56 ± 4.56		10.52 ± 1.53		7.91 ± 0.33	
2000-5000	181 (36.42)	14.84 ± 5.19		26.78 ± 3.48		24.82 ± 4.06		10.35 ± 1.31		7.79 ± 0.51	
5000-10000	137 (27.57)	14.55 ± 4.71		26.58 ± 3.25		25.23 ± 4.41		10.50 ± 1.28		7.80 ± 0.51	
10000-20000	63 (12.68)	16.56 ± 4.65		27.54 ± 4.34		26.94 ± 3.64		10.63 ± 1.30		7.89 ± 0.36	
>20000	32 (6.44)	15.88 ± 4.69		27.75 ± 4.30		26.06 ± 4.36		11.16 ± 1.65		7.91 ± 0.30	
Living with Others			0.008		0.006		0.006		0.042		0.475
Yes	448 (90.14)	15.04 ± 4.93		27.08 ± 3.66		25.41 ± 4.21		10.56 ± 1.33		7.84 ± 0.44	
No	49 (9.86)	13.02 ± 5.26		25.67 ± 3.10		23.65 ± 4.48		10.06 ± 1.70		7.78 ± 0.55	
Medical Insurance Type			0.007		0.005		0.040		0.083		0.383
Only social medical insurance	381 (76.66)	14.85 ± 4.90		27.16 ± 3.61		25.35 ± 4.23		10.49 ± 1.39		7.83 ± 0.46	
Only commercial medical insurance	28 (5.63)	13.07 ± 5.12		25.14 ± 2.93		23.29 ± 4.02		10.04 ± 1.26		7.82 ± 0.48	
Both social and commercial medical insurance	72 (14.49)	16.03 ± 5.26		26.94 ± 3.99		25.64 ± 4.24		10.75 ± 1.26		7.88 ± 0.44	
No insurance	16 (3.22)	12.38 ± 4.18		24.81 ± 2.14		24.13 ± 4.92		10.63 ± 1.54		7.75 ± 0.45	
Are you aware of the irradiation site for this treatment?			<0.001		0.009		0.045		0.015		0.081
Yes	463 (93.16)	15.14 ± 4.88		27.04 ± 3.64		25.34 ± 4.25		10.55 ± 1.34		7.84 ± 0.45	
No	34 (6.84)	10.76 ± 4.74		25.56 ± 3.26		23.85 ± 4.30		9.94 ± 1.65		7.74 ± 0.51	
Are you aware of the radiotherapy method for this treatment?			<0.001		0.011		0.039		0.002		0.352
Yes	350 (70.42)	16.10 ± 4.65		27.21 ± 3.74		25.46 ± 4.33		10.62 ± 1.38		7.84 ± 0.46	
No	147 (29.58)	11.85 ± 4.47		26.30 ± 3.30		24.71 ± 4.07		10.23 ± 1.34		7.82 ± 0.45	
Do you have a family number with medical education background?			<0.001		<0.001		0.560		0.723		0.008
Yes	108 (21.73)	17.77 ± 4.98		25.92 ± 4.07		25.44 ± 4.22		10.42 ± 1.24		7.93 ± 0.33	
No	389 (78.27)	14.03 ± 4.68		27.23 ± 3.46		25.18 ± 4.28		10.53 ± 1.41		7.81 ± 0.48	
Health Literacy Score			0.114		<0.001		0.424		0.769		0.055
≤9	396 (79.68)	15.00 ± 4.87		26.50 ± 3.31		25.39 ± 4.00		10.49 ± 1.33		7.86 ± 0.42	
>9	101 (20.32)	14.22 ± 5.42		28.68 ± 4.30		24.63 ± 5.16		10.55 ± 1.54		7.74 ± 0.58	

The mean knowledge, attitude, practice, medical information acquisition, and patient comprehensive scores were 14.84 ± 4.99 (possible range: 0 - 28), 26.94 ± 3.63 (possible range: 8 - 40), 25.24 ± 4.26 (possible range: 6 - 30), 10.51 ± 1.37 (possible range: 3 - 15), and 7.83 ± 0.46 (possible range: 0 - 10), separately. Differences in knowledge scores were more likely to be found among patients with different age, education, employment status, monthly family income, medical insurance type, and whether having a family number with medical education background. More specifically, those younger, working full time, having a higher monthly income, being aware of the irradiation site for this treatment, being aware of the radiotherapy method for this treatment, and having a family number with medical education background were more likely to have higher scores on the radiotherapy and radiation protection section. Those living in urban, having higher education, working full-time, living with others, having both social and commercial medical insurance, being aware of the irradiation site for this treatment, and being aware of the radiotherapy method for this treatment were more likely to have higher scores on the ionizing radiation section (**Table 1**, **Supplementary Table 1**).

Meanwhile, patients with different medical insurance type, whether having a family number with medical education background, and health literacy score were more likely to have different attitude scores. Participants who from different residence, had different employment status, different monthly family income, and different medical insurance type were analyzed as being more likely to have varied practice scores. Further, participants’ medical information acquisition scores may also vary due to differences in residence Finally, having a family number with medical education background was also found to have a possible influence on patients’ patient comprehensive score (all P < 0.005) (**Table 1**).

In knowledge dimension, regarding radiotherapy and radiation protection, the question with the highest proportion choosing the “Unknown” option were KI-5 with 33.60% (**Supplementary Table 2**). For radiological examinations involving ionizing radiation, 26.56% considered it quite dangerous and 4.23% were convinced that it was very dangerous (KII-6) (**Supplementary Table 3**). Notably, the patient’s attitude was not entirely positive, with 37.22% being neutral about the side effects and possible sequela of radiation therapy (A4), and 36.22% being neutral about the health problems and anxiety associated with radiation (A7) (**Supplementary Table 4**).

Patient practices tended to be proactive in general, with more than 75% of patients having a high frequency of practice (always or often) on both P1–3 and P6. Relatively, 25.55% and 29.78% had only moderate or low frequency of practice in terms of checking the hospital’s radiation safety signs (P4) and obtaining information and education provided by healthcare professionals (P5), respectively (**Supplementary Table 5**).

Correlation analyses indicated significant positive correlations between knowledge and attitude (r = 0.141, P = 0.002), as well as practice (r = 0.300, P < 0.001). Meanwhile, there was also correlation between attitude and practice (r = 0.279, P < 0.001). Besides, the medical information acquisition was found to be correlated with each dimension of KAP (all P< 0.05) (**Table 2**). The path analysis results indicated significant associations between knowledge and attitude (β = 0.142, P < 0.001), knowledge and practice (β = 0.202, P < 0.001), and between attitude and practice (β = 0.310, P < 0.001) (**Table 3**).

**Table 2:** Correlation analysis.

Dimensions	Knowledge	Attitude	Practice	Medical information acquisition	Patient comprehensive score
Knowledge	1.000				
Attitude	0.141 (P=0.002)	1.000			
Practice	0.300 (P<0.001)	0.279 (P<0.001)	1.000		
Medical Information Acquisition	0.210 (P<0.001)	0.216 (P<0.001)	0.397 (P<0.001)	1.000	
Patient Comprehensive Score	0.090 (P=0.046)	0.040 (P=0.369)	0.072 (P=0.109)	0.032 (P=0.471)	1.000

**Table 3:** Path analysis.

Characteristics	Estimate	P|
Asum <-		
Ksum	0.142	<0.001
Psum <-		
Ksum	0.202	<0.001
Asum	0.310	<0.001

## Discussion

Our study revealed a clear pattern among cancer patients: despite demonstrating inadequate knowledge and only moderate attitudes toward radiotherapy and radiation protection, they nonetheless reported proactive practices. This discrepancy highlights a critical gap between patients’ understanding and their behaviors, suggesting the influence of external support systems, such as healthcare providers. These findings underscore the importance of not only disseminating knowledge but also reinforcing trust and communication between patients and healthcare professionals. This targeted education has the potential to positively influence both attitudes and practices, thereby contributing to more informed and proactive engagement in their treatment journey.

The primary findings of the study indicate that cancer patients demonstrated inadequate knowledge, moderate attitudes, and proactive practices towards radiotherapy and radiation protection. This aligns with existing literature, which frequently highlights a knowledge gap among patients regarding their treatments (17, 18). The proactive practices observed in our study, despite the noted knowledge gap, suggest a potential area for exploration. It is plausible that patients place greater reliance on practical guidance from healthcare professionals rather than theoretical knowledge.

The findings of this study underscore the substantial impact of demographic and social factors on the knowledge, attitudes, and practices of cancer patients towards radiotherapy and radiation protection. Notably, younger patients exhibited higher knowledge scores, aligning with research suggesting that younger individuals may be more receptive to new information and adaptive in learning (19, 20). Urban residents displayed more proactive practices and greater medical information acquisition than rural and suburban residents, resonating with studies indicating that urban areas often have better access to medical information and resources (21). Patients with higher family incomes also demonstrated significantly higher knowledge and practice scores. This could be attributed to the greater accessibility to healthcare resources and information that often accompanies higher socioeconomic status (22). Similarly, those living with others had higher attitude and practice scores, possibly due to the supportive environment and shared information within households (23). The impact of educational attainment highlights not just the direct effect of education on health literacy, but also suggests varying levels of critical thinking and information processing skills among different educational groups. This calls for a diversification of patient education tools to cater to varying educational backgrounds. Socioeconomic status, as revealed through employment and income disparities, could be indicative of broader societal inequities impacting health literacy. This necessitates policies and programs addressing these systemic issues. The influence of living arrangements suggests the potential benefit of community and family-based health education interventions, leveraging the support systems to disseminate knowledge effectively. These additional insights reinforce the idea that patient education in cancer care must be multifaceted, taking into account not just demographic factors, but also cognitive, social, and systemic influences to effectively enhance patient knowledge and, subsequently, treatment outcomes (24, 25). One possible explanation for the observed proactive practices despite limited knowledge is patients’ reliance on healthcare professionals for guidance. Cancer patients often perceive their physicians as the most credible source of information and instruction, and may therefore follow recommendations closely even when their own understanding of radiotherapy and radiation protection is limited. This behavioral pattern reflects a trust-based compliance mechanism, where patients defer to professional expertise rather than acting on personal health literacy. Such dynamics highlight the need to maintain and strengthen provider-patient communication.

The correlation analyses in our study provide a nuanced understanding of how knowledge, attitudes, and practices interrelate in the context of radiotherapy and radiation protection among cancer patients. The strong positive correlation between knowledge and both radiotherapy & radiation protection and ionizing radiation suggests that increased understanding of treatment specifics is linked to better patient outcomes. Similarly, the significant associations between knowledge and attitude, practice, and medical information acquisition underscore the fundamental role of knowledge in shaping patients’ perceptions and behaviors towards their treatment. These findings echo the perspectives of health behavior theories, which posit that informed patients are more likely to exhibit positive attitudes and proactive practices (26). The path analysis results further support these associations, demonstrating significant relationships between knowledge and both attitude and practice, as well as between attitude and practice. In light of these results, healthcare providers should develop multilevel educational interventions that go beyond factual instruction. These should include interactive counseling sessions, use of visual materials, and digital reminders to reinforce both knowledge and behavioral compliance. Additionally, clinical workflows should integrate routine assessment of patient understanding and tailor communication strategies accordingly. Strengthening the educational role of the clinical encounter may not only improve knowledge but also enhance patient confidence and long-term engagement with radiation safety practices. Interventions should be designed to foster an environment where continuous learning is encouraged, and practical application of knowledge is facilitated (27, 28). Additionally, the significant role of attitude in mediating the relationship between knowledge and practice suggests that healthcare professionals should also focus on strategies that positively influence patients’ perceptions and feelings towards their treatment (29, 30).

The radiotherapy and radiation protection knowledge section of our study revealed a varied understanding of radiotherapy and radiation protection among patients. Notably, a significant number of patients were well-versed in the basic concept of radiotherapy as a therapeutic method, contrasting with the lowest awareness regarding radiation from internal sources. It is recommended that healthcare providers emphasize education about different radiation sources and their implications during treatment. Visual aids and simplified explanations could be particularly effective in enhancing understanding, especially for complex concepts like internal radiation therapy (31, 32). In the radiotherapy and ionizing radiation knowledge section, patients exhibited a high correct rate in identifying natural sources of ionizing radiation, indicating a relatively better general understanding of environmental radiation exposure. However, misconceptions were apparent in identifying medical procedures involving ionizing radiation, particularly with MRI and ultrasound, where more than 30% of patients incorrectly believed this involved ionizing radiation. This discrepancy echoes findings from Lee and Bastiani L, suggesting a need for clearer communication about medical imaging technologies (33). Educative interventions should focus on delineating the types of radiation used in different imaging techniques, possibly through brochures or digital media, to rectify these misconceptions. The findings from responses to specific questions further illustrate critical misconceptions that could compromise informed decision-making. For instance, the belief held by over 30% of patients that MRI involves ionizing radiation highlights a widespread misunderstanding of diagnostic imaging technologies. Addressing such misconceptions is crucial, as patients may experience unnecessary anxiety or make suboptimal decisions based on inaccurate information (34). Similarly, the high proportion of neutral responses regarding radiation side effects suggests ambivalence or a lack of awareness, which could affect treatment compliance. These gaps underscore the need for structured counseling that not only conveys technical knowledge but also actively engages patients in understanding potential risks and benefits. Patients’ attitudes towards radiotherapy revealed both strong agreement on its necessity and a substantial degree of neutrality regarding its side effects. This pattern of responses, showing confidence in the treatment but apprehension about its consequences. Addressing this, healthcare providers should adopt a two-pronged approach: reinforcing the importance and efficacy of radiotherapy while also openly discussing potential side effects. Tailored counseling sessions can be effective in managing patients’ concerns and expectations. Regarding practices, a majority of patients exhibited proactive behaviors, such as complying with healthcare advice and cooperating with radiation safety precautions. To further enhance this, healthcare providers could implement regular follow-up sessions to reinforce practices and provide a platform for addressing any patient concerns. The use of interactive digital tools for reminders and education could also be a beneficial supplement to traditional methods (35, 36).

This study has several limitations. Firstly, its cross-sectional design limits the ability to draw causal inferences regarding the relationships among knowledge, attitudes, and practices. Secondly, as the study was conducted exclusively at a single tertiary care center, the findings may not be generalizable to broader or more diverse cancer patient populations. Finally, the use of self-reported questionnaires may have introduced response and social desirability biases, as some participants may have misunderstood certain questions or provided answers they perceived as more acceptable, potentially affecting the accuracy of the KAP assessment. Further studies could consider a longitudinal, multi-center design using objective evaluation tools to address the limitations.

In conclusion, cancer patients demonstrated inadequate knowledge, moderate attitudes, and proactive practices regarding radiotherapy and radiation protection. It is recommended that clinicians and healthcare providers prioritize the enhancement of educational programs and resources to improve patient understanding of these aspects. This targeted approach has the potential to positively influence attitudes and practices, ultimately contributing to improved patient outcomes and adherence to treatment protocols. Future efforts should consider developing tailored educational materials that accommodate varying literacy levels and cultural backgrounds. Additionally, digital tools—such as mobile health applications, interactive videos, or online platforms—may serve as effective channels for delivering accessible and engaging radiation education.

## Data Availability

The original contributions presented in the study are included in the article/**Supplementary Material**. Further inquiries can be directed to the corresponding author/s.
