# Supplementary material for: Knowledge, attitude, and practice of cancer patients regarding radiotherapy and radiation protection
Source: Front Oncol. 2025 Jul 30;15:1432187. doi: 10.3389/fonc.2025.1432187 (PMC12344522; doi:10.3389/fonc.2025.1432187)
Supplement: Supplementary file 1 [file Table1.docx]

**Supplemental Table 1. Scores for two knowledge sections**

|  | **N (%)** | **Knowledge of radiotherapy and radiation protection** | | **Knowledge of ionizing radiation** | |
| --- | --- | --- | --- | --- | --- |
|  |  | **Score** | **P** | **Score** | **P** |
| **Total** | 497 (100.00) | 7.12 ± 3.78 |  | 7.72 ± 2.98 |  |
| **Gender** |  |  | 0.162 |  | 0.625 |
| Male | 245 (49.30) | 7.49 ± 3.97 |  | 7.59 ± 2.95 |  |
| Female | 252 (50.70) | 6.76 ± 3.57 |  | 7.84 ± 3.02 |  |
| **Age, years** | 50.89 ± 15.36 |  | 0.031 |  | 0.069 |
| ≤52 | 249 (50.10) | 7.51 ± 3.80 |  | 7.97 ± 3.09 |  |
| >52 | 248 (49.90) | 6.73 ± 3.73 |  | 7.47 ± 2.86 |  |
| **Residence** |  |  | 0.428 |  | 0.044 |
| Rural | 175 (35.21) | 7.30 ± 3.84 |  | 7.25 ± 2.92 |  |
| Urban | 283 (56.94) | 7.06 ± 3.75 |  | 8.00 ± 3.03 |  |
| Suburban | 39 (7.85) | 6.77 ± 3.81 |  | 7.80 ± 2.75 |  |
| **Education** |  |  | 0.050 |  | <0.001 |
| Junior high school and below | 136 (27.36) | 6.83 ± 4.08 |  | 7.28 ± 2.75 |  |
| High school and technical secondary school | 124 (24.95) | 6.49 ± 3.42 |  | 7.47 ± 3.04 |  |
| College | 95 (19.11) | 7.40 ± 3.93 |  | 7.64 ± 2.84 |  |
| Bachelor's degree | 109 (21.93) | 7.78 ± 3.55 |  | 7.95 ± 3.17 |  |
| Master's degree and above | 33 (6.64) | 7.73 ± 3.88 |  | 9.94 ± 2.57 |  |
| **Employment Status** |  |  | 0.003 |  | <0.001 |
| Full-time | 139 (27.97) | 7.94 ± 3.62 |  | 8.620 ± 2.88 |  |
| Part-time/Self-employed/Freelancer | 98 (19.72) | 6.72 ± 3.71 |  | 7.16 ± 3.08 |  |
| Unemployed/Laid off | 55 (11.07) | 6.95 ± 3.65 |  | 6.44 ± 2.74 |  |
| Full-time housewife/husband | 35 (7.04) | 7.29 ± 4.57 |  | 6.91 ± 2.58 |  |
| Retired | 138 (27.77) | 6.33 ± 3.73 |  | 7.94 ± 2.83 |  |
| Student | 32 (6.44) | 8.34 ± 3.50 |  | 7.63 ± 3.42 |  |
| **Monthly Income, Yuan** |  |  | 0.043 |  | 0.138 |
| <2000 | 84 (16.90) | 6.35 ± 3.86 |  | 7.29 ± 2.74 |  |
| 2000-5000 | 181 (36.42) | 7.16 ± 3.96 |  | 7.68 ± 3.01 |  |
| 5000-10000 | 137 (27.57) | 6.93 ± 3.42 |  | 7.62 ± 3.03 |  |
| 10000-20000 | 63 (12.68) | 8.30 ± 3.58 |  | 8.25 ± 3.02 |  |
| >20000 | 32 (6.44) | 7.434 ± 4.02 |  | 8.44 ± 3.12 |  |
| **Living with Others** |  |  | 0.311 |  | 0.003 |
| Yes | 448 (90.14) | 7.17 ± 3.80 |  | 7.87 ± 2.89 |  |
| No | 49 (9.86) | 6.65 ± 3.59 |  | 6.37 ± 3.53 |  |
| **Medical Insurance Type** |  |  | 0.217 |  | <0.001 |
| Only social medical insurance | 381 (76.66) | 7.05 ± 3.78 |  | 7.806 ± 2.879 |  |
| Only commercial medical insurance | 28 (5.63) | 7.93 ± 3.27 |  | 5.14 ± 3.29 |  |
| Both social and commercial medical insurance | 72 (14.49) | 7.47 ± 4.06 |  | 8.56 ± 2.89 |  |
| No insurance | 16 (3.22) | 6.00 ± 3.14 |  | 6.38 ± 2.75 |  |
| **Are you aware of the irradiation site for this treatment?** |  |  | <0.001 |  | <0.001 |
| Yes | 463 (93.16) | 7.29 ± 3.78 |  | 7.85 ± 2.93 |  |
| No | 34 (6.84) | 4.88 ± 3.15 |  | 5.88 ± 3.15 |  |
| **Are you aware of the radiotherapy method for this treatment?** |  |  | <0.001 |  | 0.014 |
| Yes | 350 (70.42) | 8.16 ± 3.64 |  | 7.94 ± 2.84 |  |
| No | 147 (29.58) | 4.65 ± 2.88 |  | 7.20 ± 3.25 |  |
| **Do you have a family number with medical education background?** |  |  | <0.001 |  | 0.068 |
| Yes | 108 (21.73) | 9.60 ± 3.63 |  | 8.17 ± 3.27 |  |
| No | 389 (78.27) | 6.43 ± 3.53 |  | 7.59 ± 2.89 |  |
| **Health Literacy Score** |  |  | 0.059 |  | 0.813 |
| ≤9 | 396 (79.68) | 7.28 ± 3.73 |  | 7.72 ± 2.99 |  |
| >9 | 101 (20.32) | 6.52 ± 3.96 |  | 7.70 ± 2.96 |  |

**Supplemental Table 2. Knowledge Section I - Radiotherapy and Radiation Protection**

|  | **Well-known** | **Heard of** | **Unknown** |
| --- | --- | --- | --- |
| **1.** **Radiotherapy (abbreviated as RT) is a therapeutic method that uses high doses of radiation to kill tumor cells and prevent their spread. In clinical practice, doctors also use low doses of radiation (such as X-rays) to observe changes inside the patient's body and collect images (such as taking X-ray images of bones and teeth). In tumor treatment, doctors select high doses of radiation.** | 28.57 | 58.35 | 13.08 |
| **2.** **The delivery methods of radiation can be divided into external beam radiation therapy (the radiation machine is outside the body, targeting the location of tumor cells in the human body) and internal radiation therapy (radiation is emitted from a source placed inside the body).** | 22.33 | 58.35 | 19.32 |
| **3.** **Three basic measures for external beam radiation protection include increasing distance, reducing time, and shielding protection.** | 20.52 | 52.52 | 26.96 |
| **4.** **External beam radiation therapy does not make patients radioactive, allowing them to interact with their families as usual.** | 29.18 | 52.72 | 18.11 |
| **5.** **When the radiation source is inside the patient's body, the patient will also generate a small amount of radiation around them.** | 17.51 | 48.89 | 33.60 |
| **6.** **Radiotherapy not only kills tumor cells or slows their growth but also affects nearby normal cells.** | 25.15 | 57.75 | 17.10 |
| **7.** **External application of radioprotective drugs can be used to reduce the sensitivity of normal tissues to radiation, increase tolerance to radiation doses, and promote the repair of radiation damage to normal tissues using certain medications.** | 21.13 | 54.93 | 23.94 |

**Supplemental Table 3. Knowledge Section II - Radiotherapy and Ionizing Radiation**

|  | **N (%)** |
| --- | --- |
| **1. There are natural sources of ionizing radiation in the world (such as rocks, soil, radon gas in the natural environment, as well as natural radioactive elements in food and cosmic rays), and we are all exposed to them.** | |
| Correct | 424 (85.31) |
| Incorrect | 73 (14.69) |
| **2.** **Which of the following imaging tests involves exposure to ionizing radiation?** | |
| a. Ultrasound examination |  |
| No | 332 (66.80) |
| Yes | 165 (33.20) |
| b. Computed Tomography (CT) scan |  |
| No | 176 (35.41) |
| Yes | 321 (64.59) |
| c. Magnetic Resonance Imaging (MRI) |  |
| No | 231 (46.48) |
| Yes | 266 (53.52) |
| d. Mammography (Breast X-ray) |  |
| No | 236 (47.48) |
| Yes | 261 (52.52) |
| **3.** **Which imaging test provides a higher radiation dose?** | |
| Chest CT scan | 236 (47.48) |
| Chest X-ray | 133 (26.76) |
| Radiation dose is the same | 128 (25.75) |
| **4.** **After which of the following tests (or even for some time after the test) does the patient still have radioactivity?** | |
| Ultrasound with contrast | 38 (7.65) |
| CT contrast-enhanced scan | 121 (24.35) |
| Particle implant therapy | 177 (35.61) |
| All of the above | 124 (24.95) |
| None of the above | 37 (7.44) |
| **5.** **Is the radiation dose the same for a thin patient (weight 60 kg) and a heavy patient (weight 100 kg) for an abdominal CT scan?** | |
| Higher for the thin patient | 64 (12.88) |
| Higher for the heavy patient | 235 (47.28) |
| About the same | 198 (39.84) |
| **6.** **Is undergoing a radiographic examination dangerous?** |  |
| Not very dangerous | 344 (69.22) |
| Quite dangerous | 132 (26.56) |
| Very dangerous | 21 (4.23) |
| **7.** **Which group of people is more at risk when undergoing radiographic examinations?** | |
| 2-year-old child | 285 (57.34) |
| 25-year-old male | 42 (8.45) |
| 25-year-old female | 21 (4.23) |
| Middle-aged adult | 10 (2.01) |
| Elderly person | 57 (11.47) |
| No difference | 82 (16.50) |

**Supplemental Table 4. Attitude Section**

| Attitude, n (%) | Strongly Agree | Agree | Neutral | Disagree | Strongly Disagree |
| --- | --- | --- | --- | --- | --- |
| 1. I believe that radiotherapy is a good treatment, and it is necessary for me to recover my health. | 212 (42.66) | 225 (45.27) | 49 (9.86) | 9 (1.81) | 2 (0.40) |
| 2. I have complete trust in my doctor, so I am willing to accept the treatment they choose for me. | 255 (51.31) | 195 (39.24) | 39 (7.85) | 5 (1.01) | 3 (0.60) |
| 3. Although I have accepted this treatment, my understanding of it is limited, and I have doubts about its effectiveness and safety. | 132 (26.56) | 190 (38.23) | 115 (23.14) | 48 (9.66) | 12 (2.41) |
| 4. The side effects and potential consequences of radiotherapy make me very fearful. | 107 (21.53) | 154 (30.99) | 185 (37.22) | 44 (8.85) | 7 (1.41) |
| 5. I believe radiation safety protection is crucial and should be taken seriously to protect my health. | 215 (43.26) | 232 (46.68) | 44 (8.85) | 5 (1.01) | 1 (0.20) |
| 6. I trust the professional judgment and advice of healthcare providers, and it is essential to rely on the safety measures they provide to protect myself and my family. | 253 (50.91) | 203 (40.85) | 39 (7.85) | 2 (0.40) | 0 (0.00) |
| 7. I always believe that radiation can cause health problems, and this makes me anxious. | 103 (20.72) | 160 (32.19) | 180 (36.22) | 46 (9.26) | 8 (1.61) |
| 8. Even if healthcare providers tell me that the treatment I am receiving won't affect my family, I still worry that I may be radioactive and could impact their health. | 97 (19.52) | 153 (30.78) | 148 (29.78) | 85 (17.10) | 14 (2.82) |
| 9. For me, the therapeutic effects of radiotherapy take precedence over the side effects, and I am willing to undergo it for a better chance at survival. | 172 (34.61) | 259 (52.11) | 58 (11.67) | 8 (1.61) | 0 (0.00) |

**Supplemental Table 5. Practice Section**

| Practice, n (%) | Always | Often | Sometimes | Rarely | Never |
| --- | --- | --- | --- | --- | --- |
| 1. Understand and comply with the hospital's safety regulations and guidelines. | 223 (44.87) | 173 (34.81) | 81 (16.30) | 18 (3.62) | 2 (0.40) |
| 2. Follow the advice and guidance of medical staff (such as maintaining distance, wearing protective clothing, etc.). | 274 (55.13) | 141 (28.37) | 66 (13.28) | 14 (2.82) | 2 (0.40) |
| 3. Cooperate with the radiation safety measures provided by the hospital. | 288 (57.95) | 130 (26.16) | 64 (12.88) | 13 (2.62) | 2 (0.40) |
| 4. Pay attention to the radiation safety signs in the hospital. | 216 (43.46) | 154 (30.99) | 94 (18.91) | 28 (5.63) | 5 (1.01) |
| 5. Receive educational materials and awareness campaigns provided by medical staff. | 192 (38.63) | 157 (31.59) | 111 (22.33) | 36 (7.24) | 1 (0.20) |
| 6. Follow medical advice to use certain medications to promote the repair of radiation damage to normal tissues. | 232 (46.68) | 157 (31.59) | 79 (15.90) | 27 (5.43) | 2 (0.40) |
